# High-Intensity Focused Ultrasound (HIFU) Focal Therapy for Localized Prostate Cancer with MRI-US Fusion Platform

**DOI:** 10.1155/2021/7157973

**Published:** 2021-12-14

**Authors:** Chi-Hang Yee, Peter Ka-Fung Chiu, Jeremy Yuen-Chun Teoh, Chi-Fai Ng, Chi-Kwok Chan, See-Ming Hou

**Affiliations:** S. H. Ho Urology Centre, Department of Surgery, Prince of Wales Hospital, The Chinese University of Hong Kong, Hong Kong

## Abstract

**Objective:**

The study aimed at investigating the outcome of prostate HIFU focal therapy using the MRI-US fusion platform for treatment localization and delivery.

**Methods:**

It is a prospectively designed case series of HIFU focal therapy for localized prostate cancer. The inclusion criteria include clinical tumor stage ≤T2, visible index lesion on multiparametric MRI less than 20 mm in diameter, absence of Gleason 5 pattern on prostate biopsy, and PSA ≤ 20 ng/ml. HIFU focal therapy was performed in the conventional manner in the beginning 50% of the series, whereas the subsequent cases were performed with MRI-US fusion platform. The primary outcome was treatment failure rate which is defined by the need of salvage therapy. Secondary outcomes included tumor recurrence in follow-up biopsy, PSA change, perioperative complications, and postoperative functional outcomes.

**Results:**

Twenty patients underwent HIFU focal ablation. HIFU on an MRI-US fusion platform had a trend of a longer total operative time than the conventional counterpart (124.2 min vs. 107.1 min, *p*=0.066). There was no difference in the mean ablation volume to lesion volume ratio between the two. The mean PSA percentage change from baseline to 6-month is more significant in the conventional group (63.3% vs. 44.6%, *p*=0.035). No suspicious lesion was seen at 6-month mpMRI in all 20 patients. Two patients, one from each group, eventually underwent radical treatment because of the presence of clinically significant prostate cancer in the form of out-of-field recurrences during follow-up biopsy. No significant difference was observed before and after HIFU concerning uroflowmetry, SF-12 score, and EPIC-26 score. It was observed that energy used per volume was positively correlated with PSA density of the patient (*r* = 0.6364, *p*=0.014).

**Conclusion:**

In conclusion, HIFU with conventional or MRI-US fusion platform provided similar oncological and functional outcomes.

## 1. Introduction

High-intensity focused ultrasound (HIFU) has been evaluated for the treatment of several benign and malignant conditions, such as uterine fibroids, thyroid nodules, and breast cancers. The first experience with the use of HIFU for prostate cancer management was reported in 1996 [1], and the initial role of HIFU was for whole-gland ablation when patients refused or were not eligible for radical prostatectomy [2, 3]. The use of HIFU for focal treatment represents a more recent concept aiming to provide the best balance between oncological control and minimizing the side effect profile. Such focal treatment extends from the initial report as a salvage therapy for focal recurrence after radiotherapy [4] to being the primary treatment for nonmetastatic prostate cancer [5, 6].

The diagnostic pathway of prostate cancer has been revolutionized by the use of mpMRI. MRI-ultrasound (US) guided prostate biopsy has been validated for its ability to increase the detection rate of clinically significant cancers with a reduction in the overdiagnosis of clinically insignificant cancers [7, 8]. While precision of prostate biopsy has been improved by the MRI-US fusion platform, such precision in lesion localization has not been incorporated in the conventional focal therapy delivery. Conventional HIFU focal therapy has been performed relying on transrectal ultrasound only. Recently, the MRI-US fusion software has been introduced to HIFU focal therapy platform in an attempt to enhance treatment precision. Our study aimed to describe the results and change in prostate parameters after MRI-US fusion HIFU focal therapy.

## 2. Materials and Methods

This was a prospective observational study on consecutive patients with localized prostate cancer who underwent HIFU focal therapy between April 2019 and September 2020. Men aged between 40 and 80 years old who were diagnosed of prostate cancer with the following criteria were considered potential candidate for HIFU focal therapy: clinically tumor stage ≤T2, visible index lesion(s) on multiparametric MRI less than 20 mm in diameter, position of tumor less than 40 mm from rectum on MRI, absence of Gleason 5 pattern on prostate biopsy, and PSA ≤ 20 ng/ml. Patients were excluded from the study if they had active urinary tract infection, if they were on anticoagulation, and if they were unfit for contrast multiparametric MRI exam. The study protocol was approved by the local institutional ethics review board (CREC 2018.556-T) and was conducted in accordance with the ethical standards of the Helsinki Declaration of 1975 and its later versions. Written informed consent was obtained from all patients for study enrolment.

All patients who entered the study had prior mpMRI of the prostate. Diagnosis of prostate cancer was either by template systematic biopsy or MRI-guided ultrasound fusion biopsy. Once the patient was identified to fulfil the aforementioned inclusion and exclusion criteria after recruitment, he would have the following assessments: TRUS (transrectal ultrasound) to assess if there is any calcification in close proximity to the lesion that would hinder HIFU energy delivery, PSA, International Prostate Symptom Score (IPSS), overactive bladder symptom score (OABSS), International Index of Erectile Function (IIEF-5), 12-item short form survey (SF-12), expanded prostate cancer index composite (EPIC-25), and uroflowmetry.

### 2.1. HIFU Treatment

HIFU therapy was performed using the Sonablate® 500 device (Sonacare Inc., USA), and treatments were delivered in a focal lesion ablation or quadrant fashion depending on the gland volume, tumor volume, and its location. Administration of HIFU focal therapy was based on the strategy of index lesion ablation, i.e., only index lesion was ablated in patients with multifocal disease when untreated areas did not exhibit any Gleason 6 lesion more than 3 mm in diameter. While conventional HIFU ablation was performed with cognitive recognition of lesion location, fusion of MRI and ultrasound images for the localization of lesion during HIFU treatments was performed with the Sonafuse™-MIM Symphony™ platform in the later 50% of the case series. The Sonafuse™-MIM Symphony™ platform adopted a similar fusion process as in bkFusion™ for prostate biopsy, with prior installation of MRI images and subsequent contouring of the gland and index lesions. Limitation of the fusion platform could be attributed to its rigid fusion instead of elastic fusion mode of operation. Ablation margin of 7 mm was adopted in the treatment. The whole treatment procedure was performed under general anaesthesia with real-time ultrasound monitoring (supplementary video). A urethral catheter was inserted during or after the procedure depending on the location of the lesion.

mpMRI was performed on postoperative day 7 and postoperative 6 months for evaluation. Routine follow-up biopsy was arranged after 6-month MRI. Prostate volume, lesion volume, and ablation zone volume were reconstructed and evaluated by OsiriX MD 12.0. Functional assessment and PSA were checked at 3 months and 6 months. The primary outcome was treatment failure rate which is defined by the need of salvage therapy. Secondary outcomes included tumor recurrence in follow-up biopsy, PSA change, perioperative complications, and postoperative functional outcomes.

Statistical analysis was performed using SPSS 26.0 (IBM Corp., Armonk, NY, USA). Postoperative changes in continuous variables were compared by the *t*-test. Comparison of more than 2 variables was performed by the one-way repeated measures ANOVA test, and linear regression was performed by the Pearson correlation test. A *p* value of <0.05 was regarded as significant.

## 3. Results

A total of 20 patients with 22 index lesions underwent HIFU focal therapy for prostate cancer over the study period (Table 1). Mean PSA level of the patients was 8.70 ± 3.5 ng/ml. Six patients had their cancer diagnosed by transrectal biopsy and 14 patients had transperineal prostate biopsy under local anaesthesia. All lesions were visible on preoperative mpMRI. The two patients with ISUP grade group 4 disease had isolated target biopsy of Gleason 4 + 4 prostate cancer at the target in an 18–24-core template. Twelve patients had lesions belonging to ISUP grade group 1, of which 10 of them had tumor volume >0.5 cm^3^ on MRI images. The other two patients with tumor volume <0.5 cm^3^ refused the option of active surveillance and agreed to HIFU focal treatment. Most lesions were located at the peripheral zone.

All patients underwent focal or quadrant ablation. No hemiablation or whole-gland ablation was performed. The decision of having urethral catheter in situ during HIFU therapy depended on the proximity of the lesion to the urethra. The first 10 patients underwent conventional HIFU focal therapy, and the later 10 patients had MRI-US fusion-guided treatment. Perioperative data of HIFU focal therapy are given in Table 2. A trend of a longer total operative time was observed for patients with fusion platform than without fusion platform (124.2 min vs. 107.1 min, *p*=0.066). No statistically significant difference was observed between the two modalities with respect to ablation volume to lesion volume ratio. In the first six cases of the series, urethral catheter was removed on postoperative day 1. Four patients failed to initiate self-void on day 1 or were readmitted soon after discharge for retention of urine, requiring reinsertion of catheter. As a result, we subsequently kept the catheter for 1 week for the rest of the patients. Among the remaining 14 patients, 3 patients failed to void on day 7, requiring another attempt to wean off catheter later. For the three patients with Clavien-Dindo grade 1 complications, 3 were retention of urine requiring readmission and 1 was haematuria managed conservatively.

mpMRI at post-HIFU 1 week revealed that ablation zone had covered region of interest in all patients. Mean percentage drop of PSA at 6-month was 44.6% in the fusion group and 63.3% in the conventional group (*p*=0.035). At 6-month mpMRI, no suspicious lesion was observed in all 20 patients. Twelve patients went on to have a follow-up biopsy. Two patients (one from fusion HIFU on a peripheral zone lesion and one from conventional HIFU on a transitional zone lesion) were found to have clinically significant prostate cancer and both of them were out-of-field recurrence (GS 4 + 4 1 mm focus and GS 4 + 5 2 mm focus). These two patients eventually received radical treatment (one with robotic radical prostatectomy and the other one with radiotherapy). The radical prostatectomy specimen yielded a pT2aN0 prostate cancer, with a GS 4 + 5 tumor focus occupying 5% of the prostate volume. Another 2 patients with nonclinically significant prostate cancer (ISUP grade group 1) out-of-field recurrence was found on follow-up biopsy. The mean 6-month PSA of those in the cohort without recurrence and those with recurrence was 4.3 ± 3.2 and 3.4 ± 1.4, respectively (*p*=0.348). The mean PSA nadir of these 2 groups was 3.6 ± 2.4 and 3.4 ± 1.4, respectively (*p*=0.096).

**Figure 1:**
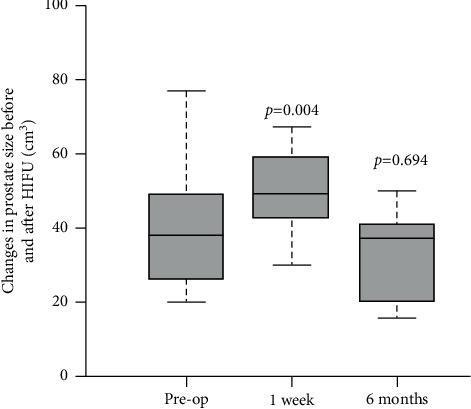
Boxplot of the change in prostate size before and after HIFU treatment. *P* value reflects the *t*-test comparison against the preoperative baseline value.

Significant increase in prostate volume was observed at postoperative 7 days compared with baseline (54.5 ± 20.1 cm^3^ vs. 39.5 ± 14.9 cm^3^, *p*=0.004). However, the mean prostate volume at 6 months (38.5 ± 21.8 cm^3^) was similar to baseline (Figure 1). PSA showed a significant drop after the surgery, with mean PSA <4 ng/ml at 6 months (Table 3). Comparison between baseline mean IPSS and post-HIFU 3-month mean IPSS showed a significant improvement (11.2 ± 5.8 vs. 8.4 ± 5.1, *p*=0.034). Preoperatively 13 patients were on alpha blocker for lower urinary tract symptoms (LUTS). Among these 13 patients, 8 patients were taken off alpha blockers at 6 months. No significant difference was observed before and after HIFU concerning uroflowmetry, SF-12 score, and EPIC-26 score. With respect to erectile function, at 3 months, a significant difference in the mean IIEF-5 score was found compared with baseline (11.7 ± 8.5 vs. 15.1 ± 6.5, *p*=0.001), yet not the same was observed with respect to the 6-month mean IIEF (13.7 ± 7.6 vs. 15.1 ± 6.5, *p*=0.314). The number of patients on phosphodiesterase type 5 inhibitor (PDE5-I) at baseline, 3 months, and 6 months was 0, 3, and 6, respectively.

**Figure 2:**
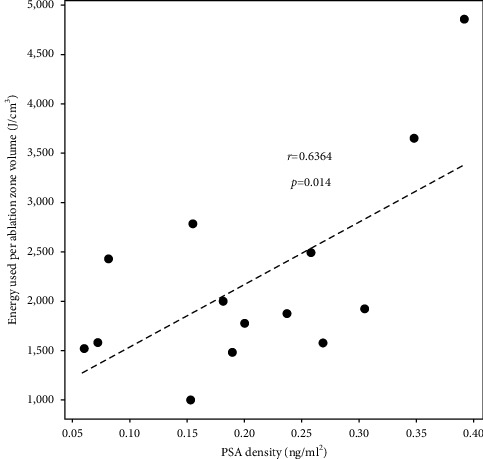
Scatter plot of the energy used per ablation zone volume in correlation to PSA density of the respective prostate gland.

Concerning the technical aspect of HIFU application, the relationship between the following factors were investigated: total energy applied, ablation zone volume, and PSA change. It was observed that energy used per volume was positively correlated with PSA density of the patient (Figure 2). However, total energy used was not correlated with PSA change (*p*=0.2431) and percentage change in PSA (*p*=0.374). Comparing the cases of HIFU focal therapy with or without fusion assistance, no difference was observed with respect to mean ablation zone volume to lesion volume ratio (62.8 and 66.4, *p*=0.473). However, the mean PSA percentage change from baseline to 6-month was more significant in the conventional group than in the fusion group (44.6 vs. 63.3, *p*=0.035).

## 4. Discussion

Focal therapy with HIFU ablation has proven to be an effective treatment option for prostate cancer with short-to-medium term results [9]. Besides oncological criteria, the size of the prostate gland also determines the outcome of treatment. The transrectal route of energy delivery by the HIFU probe may hinder access to anterior lesion [10], and the tissue swelling after HIFU treatment renders the placement of a urethral or suprapubic catheter, a common practice after the procedure [11]. Such change in prostate size has led to some centres performing transurethral resection of prostate (TURP) before or simultaneously with the HIFU procedure [12]. Previously, Shoji et al. reported the result of prostate swelling and shift after partial gland and whole-gland HIFU ablation [13]. Our study also investigated the perioperative change in prostate dimensions in focal HIFU ablation for prostate cancer by using the OsiriX MD software. A statistically significant increase in prostate volume was observed on postoperative day 7, which eventually returned to baseline at 6 months (Figure 1). This accounts for the static results at 3 months and 6 months with respect to IPSS, OABSS, EPIC-26 LUTS score, and uroflowmetry parameters (Table 3).

The availability of MRI-US-targeted prostate biopsy has made localization of prostate tumor more precise. This has encouraged focal HIFU therapy to be an alternative to the whole-gland or hemiablation treatment in the past. As a result, there is a call for a more accurate localization of the lesion during HIFU surgery. The MRI-US fusion platform for HIFU application allows the operator to plan the treatment zone with a reference location of the target tumor in sight, minimizing the potential error from cognitive fusion for the index lesion location. In the current HIFU series, a rigid MRI-US fusion platform was adopted. Taking from the experience of prostate fusion biopsy, in a systematic review by Venderink et al., the authors did not find any difference in clinically significant cancer detection between rigid and elastic registration for MRI-TRUS fusion-guided biopsy [14]. Similarly, Hale et al. [15] found no difference in registration errors between rigid and elastic registration overall, but rigid registration decreased the registration error of targets near the prostate edge, which would be a common location of tumor for HIFU ablation. Thus, a rigid MRI-US fusion platform was incorporated into our present study.

Our series have observed that with the fusion platform, there was a trend of a longer operative time without an increase in ablation time. It could be explained by the extra time consumed during mapping, fusion, and planning during treatment. By evaluating the ratio between tumor volume and eventual ablation volume, we would like to assess if the use of MRI-US fusion platform during HIFU treatment would bring about a more focal ablation. We found that the PSA drop was more significant in the conventional group, with a higher mean ablation volume to lesion volume ratio, eventhough it has not yet reached a statistically significant difference. It could be accounted by the tendency that without a clear tumor location in sight, operators tend to ablate a larger area to minimize the chance of missing any lesion. It thus results in a larger ablation volume and a bigger PSA drop after HIFU treatment. Sivaraman et al. retrospectively reviewed their cohort of HIFU patients with Ablatherm® (no fusion platform) and Focal-One® (with fusion platform) [16]. No significant difference was found with respect to perioperative complications, but the group with fusion platform had significantly lower urine leak at 3 months. In our series, no patient experienced any incontinence in both modalities. Further evaluation is needed to confirm the benefits of MRI-US fusion HIFU treatment.

Some authors have advocated the use of contrast-enhanced ultrasound to define the ablation zone after HIFU [17]. Early postoperative MRI can be another option for the same purpose. An area of hypointensity surrounded by a peripheral rim of enhancement could be observed in patients after HIFU as early as postoperative day 2 [18] to day 14 [19]. Thus, our study used MRI on day 7 for assessment of ablation zone. PSA has been commonly used for follow-up after focal therapy. Some series have adopted the Phoenix ASTRO definition as a way to document biochemical recurrence [20, 21]. On the other hand, Huber et al. found that PSA criteria have significant variability in performance characteristics to rule out or detect failure after focal HIFU therapy. Furthermore, the diagnostic performance of PSA demonstrates a wide range of accuracy metrics at different time points [22]. Our series made use of a trimodality assessment which includes PSA, MRI, and biopsy for follow-up after HIFU focal therapy. In contrast to von Hardenberg et al. who found that out of the 20 patients that had biopsies at 12 months, 8 patients had a positive biopsy within the ablation zone, and our series did not reveal any in-field recurrence [23] which was similar to Shoji et al. [21]. The two significant prostate cancers picked up by follow-up biopsy in the series could not be identified with PSA and MRI alone early in the follow-up period. The series by Ganzer et al. also demonstrated limited sensitivity of posttreatment MRI for clinically significant prostate cancer [24]. Dellabella et al. identified 38.4% of their patients having prostate cancer on 1-year biopsy, of whom 61.8% of patients went on for another line of treatment [25]. Further study is warranted to investigate the role of routine biopsy in evaluating recurrence after focal therapy for prostate cancer.

HIFU leads to tissue destruction by means of thermal and mechanical effects. Pressure fluctuations inside tissue induce hyperthermia and the mechanical effects of ultrasound results in tissue damage by cavitation and radiation force [11]. The deposition of HIFU energy brings about coagulative necrosis known as biological focal region (BFR), of which its dimension depends on the acoustic pressure, exposure time, and tissue characteristics [26]. Our series demonstrated the positive correlation between total energy used for sonication and the eventual volume of coagulative necrosis shown on MRI. Furthermore, the energy consumption per volume of coagulative necrosis is found to be correlated to the preoperative PSA density of the patient. Such observation may be explained by the differential tissue response to HIFU energy in different cases. During HIFU focal therapy session, there is continuous real-time monitoring of the thermal effects by ultrasound visualization of the intraprostatic acoustic pattern changes which include any “popcorn effect” as caused by collapse of gas bubbles. Because, in our experience, the degree of the popcorn effect was different in every case, and not every tumor ablation would result in popcorn acoustic appearance on ultrasound; there might be a difference in HIFU energy requirement between normal tissue and tumor lesions and between different tumors. Energy delivery level is adjusted by the surgeon in real-time along the whole course of HIFU treatment in order to ensure an adequate tissue response. Difference in PSA density between different prostate cancer cases can represent a difference in architecture of the tumors. Prostate cancer cells do not make more PSA [27], and the elevated serum PSA levels are a product of disruption of cellular structure within the prostate gland [28]. Taking reference from the investigation of bioimpedance of prostate tissue, bioimpedance increases with increasing distortion of gland architecture [29]. The difference in PSA density can entail a difference in tissue characteristics, implying a difference in HIFU energy requirement to deliver the same acoustic visual feedback on ultrasound.

There are limitations of our study. In our series, only 60% of the patients underwent the 12-month follow-up biopsy after HIFU treatment. While our results with respect to PSA, PHI, and MRI provided the early outcome profile of HIFU treatment, a more complete histological assessment of the cohort at 12 months would be desired. However, patient resistance to compliance with follow-up biopsy was common in the absence of rising PSA or an indication of residual disease on MRI. In the major focal HIFU therapy series in the literature, it was reported that only 37.1–52.4% patients complied to a follow-up biopsy after HIFU therapy [5, 30]. Another limitation of our study is the lack of randomization in the comparison of the results between conventional HIFU platform and MRI-US fusion HIFU platform. The results from our prospectively designed case series can be used as the basis for future randomized controlled trial to investigate the treatment outcome of the fusion HIFU platform.

## 5. Conclusion

HIFU focal therapy for prostate cancer resulted in an early tumor clearance on MRI assessment, with a significant drop in PSA and PHI after treatment. Energy consumption during HIFU therapy is positively correlated with ablation volume and tissue characteristic as reflected by PSA density. While MRI-US fusion platform for HIFU focal therapy could allow visualization of the lesion during planning and ablation, further evaluation is needed to confirm the benefits of MRI/US-fusion HIFU treatment.

## Figures and Tables

**Table 1:** Baseline characteristics of the patients.

Characteristics	
Mean age (years) ± SD	68.4 ± 5.9
Mean BMI (kg/m^2^) ± SD	23.8 ± 3.0
PSA	
<4 ng/mL (*n*)	1 (5%)
4–10 ng/mL (*n*)	13 (65%)
>10–20 ng/mL (*n*)	6 (30%)
Mode of diagnosis	
MRI-USG fusion target + systematic biopsy (*n*)	14 (70%)
Mapping transperineal systematic biopsy (*n*)	6 (30%)
Number of index lesions per HIFU surgery	
1 (*n*)	18 (90%)
2 (*n*)	2 (10%)
Characteristics of index lesions under treatment on MRI	
Visible on MRI (*n*)	22 (100%)
PI-RADS 3 (*n*)	1 (5%)
PI-RADS 4 (*n*)	14 (70%)
PI-RADS 5 (*n*)	5 (25%)
ISUP grade group of index lesions under treatment	
Grade group 1 (*n*)	12 (54.5%)
Grade group 2 (*n*)	5 (22.7%)
Grade group 3 (*n*)	3 (13.6%)
Grade group 4 (*n*)	2 (9.1%)
Grade group 5 (*n*)	0
Index lesion location	
Peripheral zone (*n*)	18 (81.8%)
Transitional zone (*n*)	4 (18.2%)
Apex (*n*)	5 (22.7%)
Middle (*n*)	9 (40.9%)
Base (*n*)	8 (36.4%)
Mean distance between the lesion from rectum (mm)	15.7 ± 8.3
Mean index lesion size (cm^3^) ± SD	0.89 ± 0.95

SD, standard deviation; BMI, body mass index; PSA, prostatic specific antigen.

**Table 2:** HIFU surgery and perioperative details.

Parameters	With fusion	Without fusion	Overall	*P* value
Mean total operative time (mins) ± SD	124.2 ± 16.9	107.1 ± 28.3	115.2 ± 24.6	0.066
Mean actual HIFU time (s) ± SD	37.4 ± 10.9	42.9 ± 13.2	40.3 ± 12.2	0.172
HIFU with urethral catheter in situ (*n*)	4	3	7 (35%)	0.639
Lesion visible on ultrasound during HIFU (*n*)	5	6	11 (55%)	0.653
Number of ablation zones used during HIFU				
1 (*n*)	0	0	0 (0%)	
2 (*n*)	0	5	5 (25%)	
3 (*n*)	10	5	15 (75%)	
Mean ablation volume to lesion volume ratio	62.8	66.4	65.1	0.473
Mean urethral catheter in situ days ± SD	8.7 ± 7.2	9.8 ± 5.7	9.3 ± 6.4	0.355
Same day discharge from the hospital (*n*)	8	3	11	0.025
90-day complication (Clavien-Dindo grade) (*n*)				
Grade 1	1	3	4	0.264
Grades 2–5	0	0	0	
Mean PSA percentage change from baseline to 6-month (%) ± SD	44.6 ± 21.0	63.3 ± 22.4	53.9 ± 23.2	0.035

PSA, prostate-specific antigen; SD, standard deviation.

**Table 3:** Overall changes in parameters after HIFU focal therapy.

Parameters	Baseline	3 months	*P* value^#^	6 months	*P* value^*∗*^
Mean PSA (ng/ml) ± SD	8.4 ± 3.3	4.2 ± 3.1	<0.001	3.7 ± 2.4	<0.001
Mean IPSS ± SD	11.2 ± 5.8	8.4 ± 5.1	0.034	8.8 ± 5.8	0.122
Mean QoL ± SD	2.4 ± 1.3	2.1 ± 1.2	0.281	2.5 ± 1.0	0.377
Mean OABSS ± SD	4.5 ± 2.6	4.2 ± 1.9	0.325	4.6 ± 2.2	0.500
Mean IIEF-5 ± SD	15.1 ± 6.5	11.7 ± 8.5	0.001	13.7 ± 7.6	0.314
Mean SF-12 ± SD					
Physical score	48.4 ± 5.9	49.7 ± 7.4	0.304	49.3 ± 6.5	0.346
Mental score	47.6 ± 10.8	49.0 ± 7.7	0.339	48.3 ± 7.0	0.422
Mean EPIC-26 score ± SD					
Incontinence	91.3 ± 13.7	90.0 ± 18.6	0.426	90.5 ± 15.1	0.449
LUTS	81.7 ± 15.2	86.7 ± 12.2	0.165	85.8 ± 11.7	0.204
Bowel function	87.8 ± 14.6	88.4 ± 13.3	0.456	85.1 ± 17.1	0.330
Sexual function	49.6 ± 30.5	43.3 ± 30.5	0.301	40.8 ± 31.0	0.237
Hormonal function	97.5 ± 6.6	97.2 ± 5.8	0.444	97.2 ± 4.8	0.440
Uroflowmetry					
*Q*max (ml/s) ± SD	14.9 ± 6.3	14.6 ± 5.2	0.451	14.4 ± 6.9	0.428
PVRU (ml)	68.6 ± 41.1	53.2 ± 48.1	0.194	47.5 ± 42.2	0.104

SD, standard deviation; PSA, prostatic specific antigen; PHI, prostate health index; IPSS, International Prostate Symptom Score; QoL, quality of life; OABSS, overactive bladder symptom score; IIEF-5, International Index of Erectile Function; SF-12, 12-item short form survey; EPIC-25, expanded prostate cancer index composite; LUTS, lower urinary tract symptoms; *Q*max, maximal voiding velocity; PVRU, postvoid residual urine. ^#^Comparison of 3-month data against the baseline value. ^*∗*^Comparison of 6-month data against the baseline value.

## Data Availability

The data used to support the findings of this study are available from the corresponding author upon request.

## Supplementary Materials

Supplementary Materials A video clip demonstrating the technique of HIFU with MRI-USG fusion platform.
